# Epidemiological Evidence for Fecal-Oral Transmission of Murine *Kobuvirus*

**DOI:** 10.3389/fpubh.2022.865605

**Published:** 2022-04-19

**Authors:** Yuhan Gao, Wenqiao He, Jiaqi Fu, Yongzhi Li, Huan He, Qing Chen

**Affiliations:** Guangdong Provincial Key Laboratory of Tropical Disease Research, Department of Epidemiology, School of Public Health, Southern Medical University, Guangzhou, China

**Keywords:** genetic characteristic, *Kobuvirus*, murine rodent, phylogenetic analysis, prevalence, transmission route

## Abstract

**Background:**

Murine *Kobuvirus* (MuKV) is a novel picornavirus of the genus *Kobuvirus*, and was first identified in the feces of murine rodents in the USA in 2011. There is limited information on the transmission route of MuKV. Thus, we conducted a study to investigate virus detection rates in fecal, serum, throat, and lung tissue samples from murine rodents.

**Results:**

A total of 413 fecal samples, 385 lung samples, 269 throat swab samples, and 183 serum samples were collected from 413 murine rodents (*Rattus norvegicus, Rattus tanezumi*, and *Rattus rattus*) captured in urban Shenzhen. Kobuviruses were detected via RT-PCR. Only fecal samples were positive, with prevalence rates of 34.9% in *Rattus norvegicus* and 29.4% in *Rattus tanezumi*. Phylogenetic analysis based on partial 3D and complete VP1 sequence regions indicated that all of the MuKV sequences obtained belonged to *Aichivirus A*, and were genetically closely related to other MuKVs reported in China, Hungary, and the USA. Twenty-eight full-length MuKV sequences were acquired. Phylogenetic analysis of two sequences randomly selected from the two species (SZ59 and SZ171) indicated that they shared very high nucleotide and amino acid identity with one another (94.0 and 99.3%, respectively), and comparison with human *Kobuvirus* revealed amino acid identity values of ~80%. Additionally, a sewage-derived sequence shared high similarity with the rat-derived sequences identified in this study, with respective nucleotide and amino acid identity values from 86.5 and 90.7% to 87.2 and 91.1%.

**Conclusion:**

The results of the current study provide evidence that murine *Kobuvirus* is transmitted via the fecal-oral route.

## Introduction

Kobuviruses belong to the genus *Kobuvirus*, within the family *Picornaviridae*, and they are small (27–30 nm diameter), spherical, non-enveloped, positive-sense, single-stranded RNA viruses (1). They all share a common genome organization, with a length of 8.2–8.4 kb. The genome consists of a 5′ untranslated region (UTR), a single open reading frame (ORF) that encodes a large polyprotein of 2,400–2,500 amino acids which is divided into a leader (L) protein, three structural proteins (VP0, VP3, and VP1), seven non-structural proteins (2A−2C and 3A−3D), a 3′UTR, and a poly(A) tail (2). Of these, the VP1 protein is the most variable, whereas the 3D gene is highly conserved (3).

*Kobuvirus* was first isolated from a fecal sample from a patient with acute gastroenteritis in Japan in 1989 (4). Kobuviruses are widespread worldwide, and are associated with gastroenteritis, respiratory infections, and other clinical symptoms (5). They have been found in a wide range of hosts including humans (4), cattle (6), sheep (7), pigs (8), dogs (9), cats (10), foxes (11), ferrets (12), goats (13), rabbits (14), bats (15), mice (16), and rats (17). They can also be detected in the environment, such as in sewage (18). The International Committee on Taxonomy of Viruses has classified the genus *Kobuvirus* into six species; *Aichivirus A* (AiV A; formerly Aichi virus), *Aichivirus B* (AiV B; formerly bovine *Kobuvirus*), *Aichivirus C* (AiV C; formerly porcine *Kobuvirus*), *Aichivirus D* (AiV D; formerly *Kagovirus 1*), *Aichivirus E* (AiV E; formerly rabbit *Kobuvirus*), and *Aichivirus F* (AiV F; formerly bat *Kobuvirus*), and 20 genetic types (19). Human *Kobuvirus*, canine *Kobuvirus*, murine *Kobuvirus* (MuKV), feline *Kobuvirus*, and roller *Kobuvirus* belong to *Aichivirus A* (20).

Rodents are considered potential reservoirs of several novel pathogens including *Kobuvirus*. MuKV was first detected in *Peromyscus crinitus* and *Peromyscus maniculatus* feces in the USA in 2011 (16). MuKVs were subsequently found in Vietnam (17), Hungary (21, 22), and the USA (23, 24) in stool samples from various murine species including *Rattus norvegicus, Rattus tanezumi, Rattus argentiventer, Rattus losea, Rattus exulans, Mus musculus*, and *Bandicota indica*. We recently conducted studies using fecal samples to determine the circulation of MuKV from *Rattus norvegicus* in Guangdong, China (25), and investigated the prevalence and genetic characterization of MuKV in *Rattus losea, Rattus tanezumi*, and *Rattus norvegicus* in several regions in southern China (26). Related research was limited to fecal samples, however, and detailed data pertaining to *Kobuvirus* in different samples of several rodent species was lacking.

Multiple studies have shown that kobuviruses can cause gastroenteritis in humans and other animal species, but there is limited information on *Kobuvirus* transmission routes. In humans *Kobuvirus* has been detected in feces and serum samples (4, 27). Porcine *Kobuvirus* viraemia has been reported (28), and a seroprevalence study in dogs and cats suggests that high percentages of these animals contain IgG antibodies against *Aichivirus A* (29). MuKV has been found in lung, brain, heart, and liver samples from rodents (23, 24), and canine *Kobuvirus* was identified in the brain, lungs, tonsils, and liver of the same puppy (30), indicating that kobuviruses might have a broad tissue tropism. To date the transmission route of MuKV in murine rodents remains unknown. To expand knowledge of the transmission route of kobuviruses, the current study investigated detection rates of MuKV in fecal, serum, throat, and lung tissue samples from murine rodents.

## Materials and Methods

### Sample Collection

**Figure 1:**
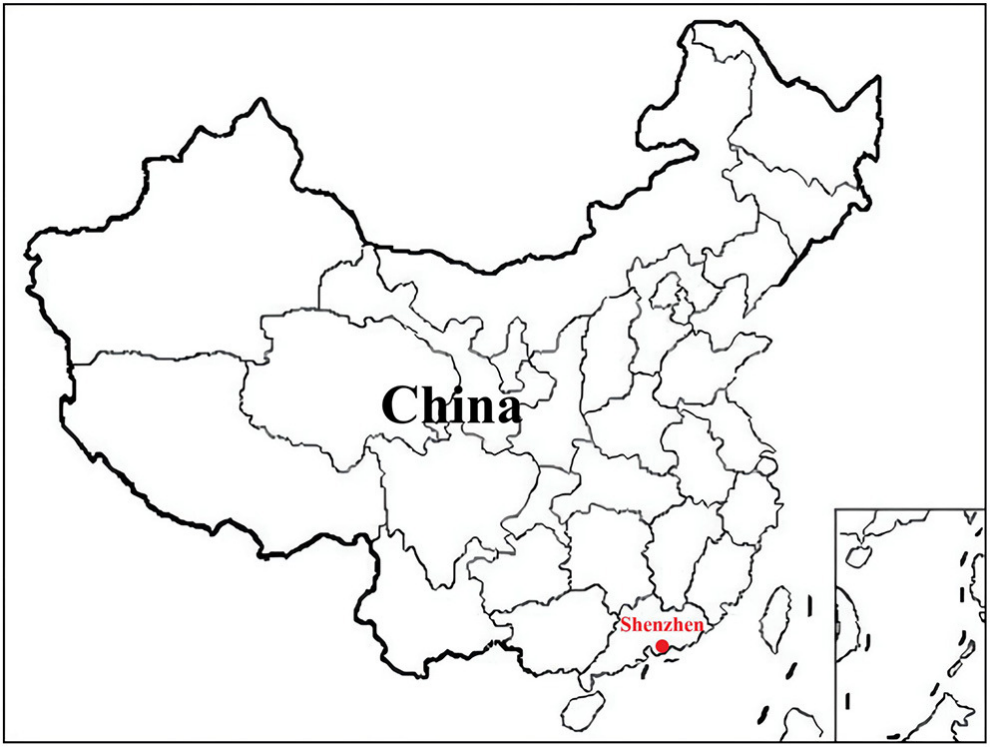
Location of the trapping site of the murine rodents in China. Map source: https://image.so.com/view?q=%E4%B8%AD%E5%9B%BD%E5%9C%B0%E5%9B%BE&src=srp&correct=%E4%B8%AD%E5%9B%BD%E5%9C%B0%E5%9B%BE&ancestor=list&cmsid=50712c46325496ee4c7fbfbded18f06e&cmras=0&cn=0&gn=0&kn=50&crn=0&bxn=20&fsn=130&cuben=0&pornn=0&manun=50&adstar=0&clw=233#id=812433445ba11b77cba553308beab1d9&currsn=0&ps=136&pc=136.

In total, 413 fecal samples, 385 lung samples, 269 throat samples, and 183 serum samples were collected from 413 murine rodents captured between October 2020 and October 2021 in Shenzhen, Guangdong Province, China (Figure 1). Urban rats were trapped once a month, using live capture traps (also known as cage traps) in residential areas, city parks, composite market, and bus station.

Sample collection was conducted in the sterile room. The rodents were anesthetized via 3% diethyl ether inhalation, and the dosage of diethyl ether was adjusted according to their heart rate, respiratory frequency, corneal reflection, and extremity muscle tension. Trained personnel wore filtering facepiece respirators, chemical safety goggles, anti-static uniforms, and chemical protective gloves to protect themselves from diethyl ether. Before animals were killed by cervical dislocation, blood was drawn via cardiac puncture and centrifuged to obtain serum samples. Individual fresh fecal and throat samples were immediately placed in RNase-free tubes with 1 mL phosphate-buffered saline (0.3% homogenate), and lung tissue samples were stored in RNAlater (Invitrogen, CA, USA). All specimens were stored at −80°C prior to further processing. The species of the trapped animals were determined by morphological identification and cytochrome B gene sequencing (31).

### Nucleic Acid Extraction and cDNA Synthesis

All samples were centrifuged at 8,000 rpm for 10 min at 4°C prior to collecting the supernatants. RNA and DNA were extracted from 200 μL of each supernatant using the MiniBEST Viral RNA/DNA Extraction Kit (TaKaRa, Kusatsu, Japan) in accordance with the manufacturer's instructions. The extracted RNA was reverse transcribed into cDNA using a Transcriptor First Strand cDNA Synthesis Kit (Roche, Basel, Switzerland). The cDNA was used directly as a template for polymerase chain reaction (PCR) assays, or stored at −20°C prior to further analysis.

### Molecular Detection of Murine *Kobuvirus*

Kobuviruses were detected via RT-PCR based on a previously described pair of consensus primers, UNIV-kobu-F/UNIV-kobu-R which amplifies a 216-nucleotide fragment of the conserved 3D region of all *Kobuvirus* species, encoding the RNA-dependent RNA polymerase (32). Among the 3D gene-positive samples, an 831-bp region of the VP1 gene was amplified via nested PCR using the *Kobuvirus*-VP1-os/oa and *Kobuvirus*-VP1-is/ia primer pair (17). The primer sequences are shown in Table 1. The PCR conditions were 94°C for 3 min, 40 cycles of 94°C for 30 s, 56°C for 1 min, and 72°C for 1 min, followed by a final extension at 72°C for 10 min. The amplified products were analyzed using 1.5% agarose gels, and visualized under ultraviolet light. Positive samples were sent to the Beijing Genomics Institute (Guangzhou, China) for sequencing.

**Table 1:** Prevalence of *Kobuvirus* in fecal, lung, throat, and serum samples of rodents (*n*, %).

**Sample**	**Family**	**Species**	**Number of samples**	**Prevalence**	***p*** **-value**
Fecal	*Muridae*	*Rattus norvegicus*	395	(138) 34.9	0.687
		*Rattus tanezumi*	17	(5) 29.4	
		*Rattus rattus*	1	0	
		Total	413	(143) 34.6	
Lung	*Muridae*	*Rattus norvegicus*	369	0	–
		*Rattus tanezumi*	15	0	
		*Rattus rattus*	1	0	
		Total	385	0	
Throat	*Muridae*	*Rattus norvegicus*	258	0	–
		*Rattus tanezumi*	11	0	
		Total	269	0	
Serum	*Muridae*	*Rattus norvegicus*	173	0	–
		*Rattus tanezumi*	10	0	
		Total	183	0	

### Genome Sequencing

Based on five murine *Kobuvirus* genome sequences from GenBank (accession numbers MF352432, MN116647, MN648600, MN648601, and MF175074), 13 pairs of primers designed to amplify the full-length viral genomes were generated (Supplementary Table 1). After sequencing, the nucleotide sequences were edited, validated, and assembled using Lasergene SeqMan software (DNASTAR, Inc. Madison, WI, USA).

### Sequence and Phylogenetic Analyses

Sequences that were newly obtained in this study were aligned with *Kobuvirus* reference sequences from the GenBank database using the ClustalW multiple sequence alignment program in MEGA (version 7.0; Oxford Molecular Ltd., UK). To analyze evolutionary relationships, phylogenetic trees were generated based on the gene sequences obtained and representative gene sequences of murine *Kobuvirus*, rat *Kobuvirus*, mouse *Kobuvirus*, sewage *Kobuvirus*, canine *Kobuvirus*, feline *Kobuvirus*, human *Kobuvirus*, ovine *Kobuvirus*, bovine *Kobuvirus*, porcine *Kobuvirus*, bat *Kobuvirus*, and rabbit *Kobuvirus*, using the neighbor-joining (NJ) method in MEGA, with 1,000 bootstrap replicates. The single open reading frame (ORF) was identified using ORF finder (https://www.ncbi.nlm.nih.gov/orffinder/), and potential proteolytic cleavage sites were predicted primarily based on the amino acid alignments of relevant sequences of kobuviruses. The GC content and nucleotide/amino acid identities among all sequences were calculated using the Sequence Identity Matrix program in BioEdit (version 7.2.5). Amino acid heterogeneity analysis was performed by aligning amino acids using DNAMAN (version 9.0). Similarity plot analysis of the almost full-length genome was conducted using SimPlot 3.5.1 software.

### Statistical Analysis

Differences in *Kobuvirus* detection rates in the different species of animals, different seasons, and sexes were assessed using chi-square tests via Statistical Product and Service Solutions software (SPSS, version 25.0; IBM Corp., Armonk, NY, USA), with associations considered statistically significant at *p* < 0.05.

### Ethics Statement

The study protocol was approved by the Animal Ethics and Welfare Committee of the School of Public Health, Southern Medical University, China, and adhered to the Rules for the Implementation of Laboratory Animal Medicine (1998) from the Ministry of Health, China. All surgical procedures were performed under anesthesia to minimize suffering. No endangered or protected species were involved in the study.

## Results

### Detection of Murine *Kobuvirus*

A total of 413 animals were trapped. Fecal, lung, throat swab, and serum samples were collected from 395 *Rattus norvegicus*, 17 *Rattus tanezumi*, and 1 *Rattus rattus* (Table 1). According to RT-PCR and nested PCR, 34.6% (143/413) of fecal specimens were positive for *Kobuvirus* RNA. Among them, the prevalence of *Kobuvirus* in different species of animals ranged from 0.0 to 34.9%, and there was no statistical difference in the positive rates of fecal samples from different species (χ^2^ = 0.751, *p* = 0.687) and sexes (χ^2^ = 0.004, *p* = 0.948; Tables 1, 2). However, the difference among seasons was significant (χ^2^ = 17.857, *p* < 0.001), and the prevalence in winter was significantly higher than other seasons (Table 2).

**Table 2:** Characteristics of prevalence kobuviruses in fecal samples of rodents (*n*, %).

**Characteristic**	**Number of samples**	**Prevalence**	***p*** **-value**
**Season**			
Spring (March–May)	109	40 (36.7)	
Summer (June–August)	63	16 (25.4)	<0.001
Autumn (September–November)	188	56 (29.8)	
Winter (December–February)	53	31 (58.5)	
Total	413	143 (34.6)	
Sex^*^			
Male	206	73 (35.4)	
Female	179	64 (35.8)	0.948
Total	385	137 (35.6)	

* *Only 385 rodents were recorded for sex*.

### Phylogenetic and Amino Acid Heterogeneity Analyses of 3D and VP1 Region Sequences

**Figure 2:**
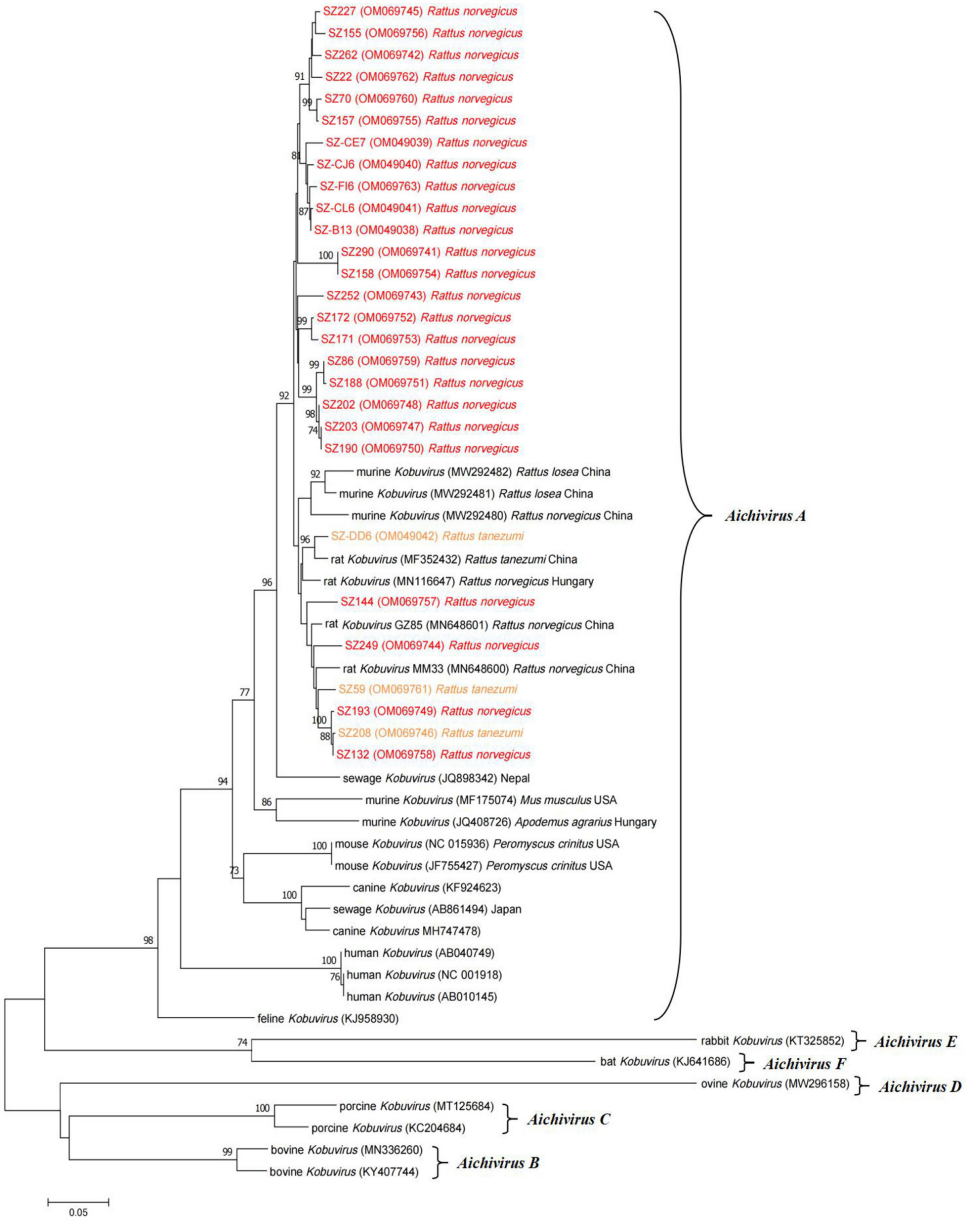
Phylogenetic tree of *Kobuvirus* based on the partial 3D nucleotide sequences (634 bp, 7,311–7,938 nt). The tree was generated by the neighbor-joining method with 1,000 bootstrap replicates, and the statistics values >70% are displayed above the tree branches. The sequences from samples collected in this study have been labeled with color. SZ, Shenzhen city in Guangdong province.

Neighbor-joining phylogenetic analysis based on partial 3D nucleotide sequences (634 nucleotides) was conducted using representative sequences detected in the study and other *Kobuvirus* reference sequences from GenBank (Figure 2). Sequences isolated from two different rodent species were highly similar, with respective nucleotide and amino acid identities of 92.6–100.0% and 99.0–100.0%. This demonstrates that the murine *Kobuvirus* strains from the same species were relatively clustered together, and the sequences identified in the study clustered with murine kobuviruses previously found in China and Hungary, within the *Aichivirus A* group, which also includes human *Kobuvirus*, canine *Kobuvirus*, feline *Kobuvirus*, and sewage *Kobuvirus*.

**Figure 3:**
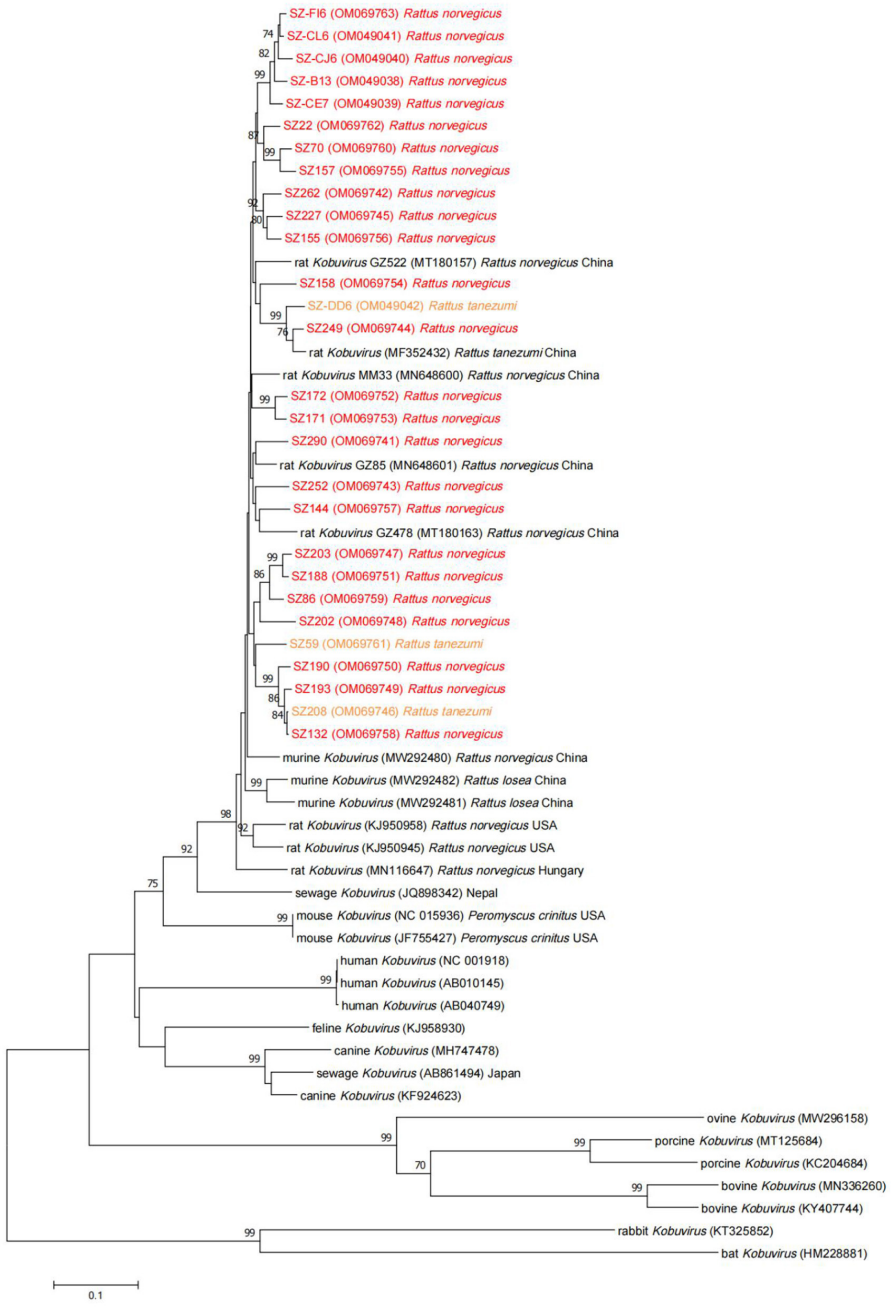
Neighbor-joining tree for *Kobuvirus* nucleotide sequences based on the complete VP1 region nucleotide sequences (831 bp, 3,012–3,842 nt). Bootstrap support for branches (1,000 replications) is indicated, and the statistics values >70% are displayed above the tree branches. Murine *Kobuvirus* (MuKV) sequences obtained in this study are represented by color. SZ, Shenzhen city in Guangdong province.

Complete VP1 sequences were obtained from samples positive for the partial 3D gene using nested PCR, and several representative sequences were selected to generate a phylogenetic tree (Figure 3) along with VP1 reference sequences from *Kobuvirus* strains in GenBank. The VP1 sequences of murine kobuviruses identified in the study shared respective nucleotide and amino acid identities of 89.8–98.9% and 97.8–100.0%. Phylogenetic analysis based on the VP1 nucleotide sequences indicated that the sequences identified in the study were more closely related to murine *Kobuvirus* strains and clustered within a major group, but origin-specific lineages of species were not apparent. Further analysis of amino acid heterogeneity and the DNAMAN output revealed that the sequences identified in the study were highly similar in 3D and VP1 regions. Compared with several reference sequences of other species, the VP1 region had more amino acid deletions and substitutions than the 3D region, with respective identities of 77.4 and 92.0% (Supplementary Figure 1).

### Genomic and Phylogenetic Analyses of Complete Sequences

Twenty-eight full-length murine *Kobuvirus* genomes were successfully sequenced from *Kobuvirus*–positive samples, including 25 from *Rattus norvegicus* and 3 from *Rattus tanezumi* (Table 3). Multiple sequence alignments were performed with these sequences, and they shared 93.1–99.0% nucleotide and 93.3–99.1% amino acid identities with each other. Two sequences (SZ59 and SZ171) were then randomly selected from the two species to further analyze the genomic structure of murine *Kobuvirus*. The SZ59 *Kobuvirus* genome was 8,252 nucleotides in length and contained a 725-nucleotide 5′-UTR, a 237-nucleotide 3′-UTR, and an ORF (7,290 nucleotides) encoding a potential polyprotein of 2,429 amino acids, excluding the poly-(A) tail. The complete genome of SZ171 had 8,250 nucleotides, and contained a 724-nucleotide 5′-UTR and a 236-nucleotide 3′-UTR. We predicted the genome organization and potential cleavage sites for the complete polyprotein genes of SZ59 and SZ171, which were identical to other previously described murine *Kobuvirus* strains. The L protein was 492 nucleotides (164 amino acids) in length, whereas the complete P1, P2, and P3 regions were 2625 nucleotides (875 amino acids), 1,833 nucleotides (611 amino acids), and 2,340 nucleotides (779 amino acids) long, respectively. The two sequences yielded GC content of 55.9–56.3% (19.5–19.8% A, 21.1–21.3% G, 24.2–24.3% T, and 34.9–35.1% C), like other kobuviruses (52–59%), which is characteristic of the family *Picornaviridae* (11).

**Table 3:** Full-length murine *Kobuvirus* (MuKV) strains identified in this study.

**No**.	**Virus** **strain**	**GenBank** **accession No**.	**Host species**	**Sample**	**Length (nt)**	**ORF**	
						**Location (nt)**	**Length (nt/aa)**
1	SZ-B13	OM049038	*Rattus norvegicus*	Feces	8,239	720–8,012	7,293/2,430
2	SZ-CE7	OM049039	*Rattus norvegicus*	Feces	8,351	728–8,020	7,293/2,430
3	SZ-CJ6	OM049040	*Rattus norvegicus*	Feces	8,220	694–7,986	7,293/2,430
4	SZ-CL6	OM049041	*Rattus norvegicus*	Feces	8,410	724–8,016	7,293/2,430
5	SZ-DD6	OM049042	*Rattus tanezumi*	Feces	8,337	477–7,769	7,293/2,430
6	SZ-FI6	OM069763	*Rattus norvegicus*	Feces	8,254	727–8,016	7,290/2,429
7	SZ22	OM069762	*Rattus norvegicus*	Feces	8,192	666–7,955	7,290/2,429
8	SZ59	OM069761	*Rattus tanezumi*	Feces	8,252	726–8,015	7,290/2,429
9	SZ70	OM069760	*Rattus norvegicus*	Feces	8,251	725–8,014	7,290/2,429
10	SZ86	OM069759	*Rattus norvegicus*	Feces	8,253	725–8,014	7,290/2,429
11	SZ132	OM069758	*Rattus norvegicus*	Feces	8,251	725–8,014	7,290/2,429
12	SZ144	OM069757	*Rattus norvegicus*	Feces	8,253	727–8,016	7,290/2,429
13	SZ155	OM069756	*Rattus norvegicus*	Feces	8,250	725–8,014	7,290/2,429
14	SZ157	OM069755	*Rattus norvegicus*	Feces	8,252	727–8,016	7,290/2,429
15	SZ158	OM069754	*Rattus norvegicus*	Feces	8,213	687–7,976	7,290/2,429
16	SZ171	OM069753	*Rattus norvegicus*	Feces	8,250	725–8,014	7,290/2,429
17	SZ172	OM069752	*Rattus norvegicus*	Feces	8,316	726–8,015	7,290/2,429
18	SZ188	OM069751	*Rattus norvegicus*	Feces	8,239	713–8,002	7,290/2,429
19	SZ190	OM069750	*Rattus norvegicus*	Feces	8,250	725–8,014	7,290/2,429
20	SZ193	OM069749	*Rattus norvegicus*	Feces	8,250	725–8,014	7,290/2,429
21	SZ202	OM069748	*Rattus norvegicus*	Feces	8,335	729–8,018	7,290/2,429
22	SZ203	OM069747	*Rattus norvegicus*	Feces	8,252	727–8,016	7,290/2,429
23	SZ208	OM069746	*Rattus tanezumi*	Feces	8,251	726–8,015	7,290/2,429
24	SZ227	OM069745	*Rattus norvegicus*	Feces	8,250	727–8,016	7,290/2,429
25	SZ249	OM069744	*Rattus norvegicus*	Feces	8,250	725–8,014	7,290/2,429
26	SZ252	OM069743	*Rattus norvegicus*	Feces	8,255	725–8,017	7,293/2,430
27	SZ262	OM069742	*Rattus norvegicus*	Feces	8,251	726–8,015	7,290/2,429
28	SZ290	OM069741	*Rattus norvegicus*	Feces	8,254	726–8,015	7,290/2,429

The predicted protease-cleavage sites of the sequences identified in the current study and reference kobuviruses are shown in Supplementary Table 2, including Q/G, P/Q, Q/H, Q/S, Q/T, Q/A, Q/P, Y/V, Q/C, and A/T. The predicted protease cleavage sites between L and VP0 (Q/G), 2A and 2B (Q/G), 2B and 2C (Q/G), and 3C and 3D (Q/S) are conserved among kobuviruses isolated from different species. In comparison, the protease-cleavage site between 2C and 3A is Q/G for all the *Kobuvirus* sequences except for ovine *Kobuvirus* (A/T). The protease cleavage sites of the sequences identified in the current study are highly conserved, and are similar to those in other species.

**Figure 4:**
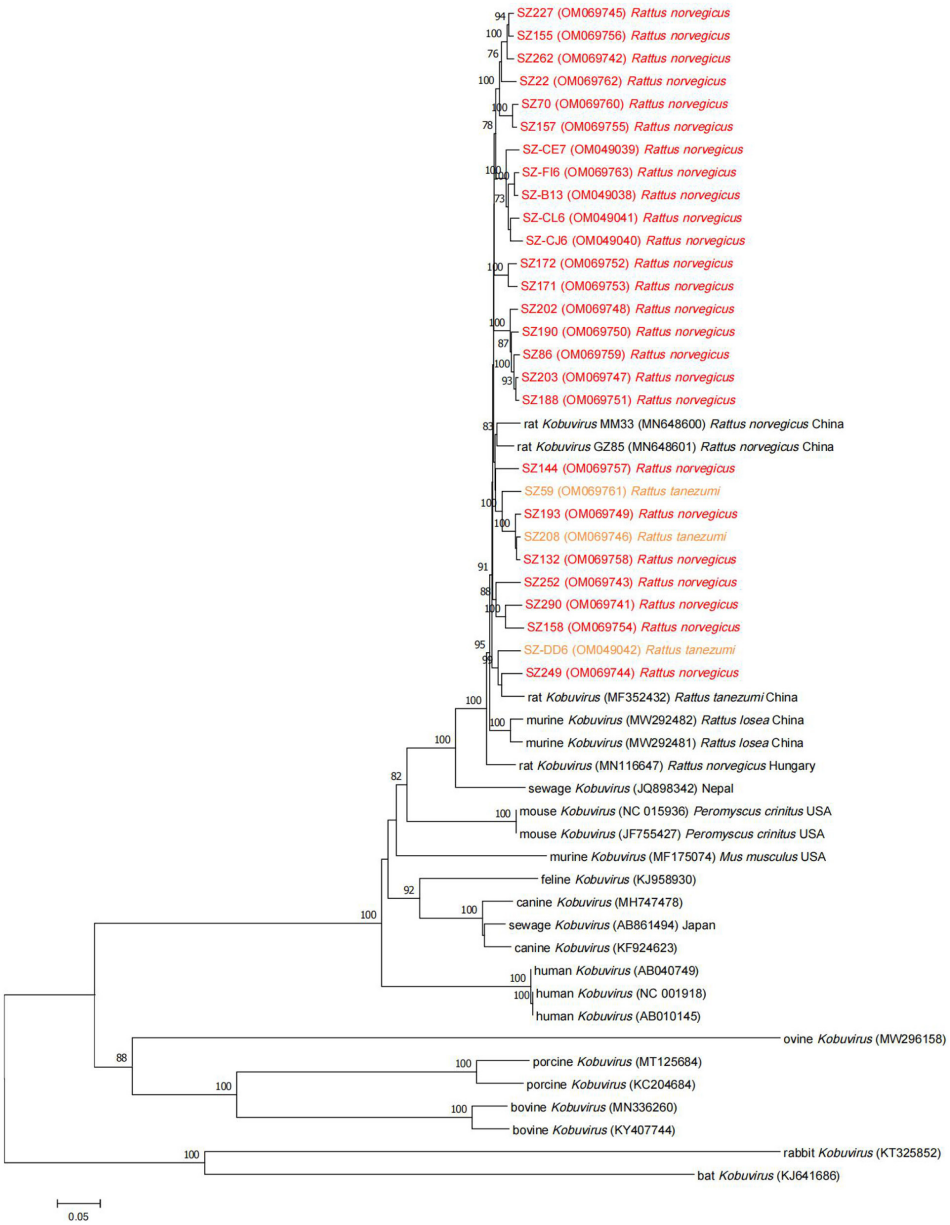
Phylogenetic tree of *Kobuvirus* based on the complete nucleotide sequences from different strains. The tree was generated by the neighbor-joining method with 1,000 bootstrap replicates, and the statistics values >70% are displayed above the tree branches. Murine *Kobuvirus* (MuKV) sequences identified in the present study are indicated by color. SZ, Shenzhen city in Guangdong province.

Phylogenetic analysis based on the complete nucleotide sequences indicated that the sequences identified in the study shared higher nucleotide (93.7–95.6%) and amino acid (94.6–96.8%) identities with two other Chinese strains (MM33 and GZ85) (25) compared to other murine *Kobuvirus* strains. All murine *Kobuvirus* strains were clustered according to their geographical regions, albeit with limited sequence support (Figure 4). A sewage-derived *Kobuvirus* sequence (JQ898342) detected in Nepal (33), which was identified as a novel *Kobuvirus* in the *Picornaviridae* family and was named *Kobuvirus* sewage Kathmandu (KoV-SewKTM) after the location of its discovery, was clustered together with the rat-derived sequences identified in this study, with high respective nucleotide and amino acid identity values of 86.5 and 87.9% to 87.4 and 89.1%. A new *Kobuvirus* (34) closely related to canine *Kobuvirus* in sewage samples that was found in Aichi Prefecture, Japan, clustered within the *Aichivirus A* group. Comparisons between the full polyprotein and L/P1/P2/P3 fragments of the two representative sequences and reference *Kobuvirus* sequences from several other species and the environment are shown in Table 4. SZ59 shared 94.0% nucleotide identity and 99.3% amino acid identity with SZ171. Compared with other *Kobuvirus* reference strains, SZ59 and SZ171 had 75.9–76.0% nucleotide identities and 80.7% amino acid identities with the human *Kobuvirus* strain AB010145, and shared the highest sequence homologies with the murine *Kobuvirus* strain MN648601 at the nucleotide (93.3–93.6%) and amino acid (98.1–98.3%) levels. In the P1, P2, and P3 regions, SZ59 and SZ171 kobuviruses exhibited more than 74% nucleotide and 77% amino acid identity with human, rat, feline, canine, and untreated sewage-derived kobuviruses, which belong to *Aichivirus A*.

**Table 4:** Nucleotide and putative amino acid sequence identities of the murine kobuviruses (SZ59 and SZ171) with reference *Kobuvirus* strains from several species and the environment.

**Gene region**	**Rat**			**Human**	**Feline**	**Canine**	**Ovine**	**Porcine**	**Bovine**	**Untreated sewage**
	**SZ59**	**SZ171**	**MN648601**	**AB010145**	**KJ958930**	**MH747478**	**MW296158**	**KC204684**	**MN336260**	**JQ898342**
**SZ59**										
L	–	93.4/97.5	92.6/95.7	64.9/63.3	56.8/44.5	57.6/50.5	35.6/21.1	39.0/20.8	42.0/26.9	–
P1	–	93.2/99.3	93.8/99.3	74.1/77.3	74.2/79.0	74.5/79.7	53.4/46.0	55.5/51.9	55.0/49.8	81.1/81.8
P2	–	95.1/99.8	95.4/100.0	77.6/82.8	78.6/84.9	80.0/85.5	52.6/45.4	57.1/58.7	63.3/62.9	87.7/94.1
P3	–	94.3/99.4	92.3/95.7	79.7/87.4	79.5/85.6	81.5/86.6	58.0/52.8	63.4/64.0	64.1/63.2	87.2/90.7
Polyprotein	–	94.0/99.3	93.6/98.1	75.9/80.7	75.7/80.2	76.8/81.4	51.9/46.1	56.6/54.9	57.2/55.2	79.3/82.2
**SZ171**										
L	93.4/97.5	–	91.8/96.9	64.5/62.2	58.4/44.5	57.2/50.0	36.5/21.1	40.2/21.8	42.0/26.4	–
P1	93.2/99.3	–	93.9/99.5	74.0/77.5	74.2/79.0	74.4/79.7	54.4/45.9	55.4/52.0	54.9/49.7	80.9/81.8
P2	95.1/99.8	–	95.1/99.8	78.5/82.8	79.1/85.1	80.1/85.7	51.9/45.4	57.6/58.7	63.5/62.9	87.9/94,2
P3	94.3/99.4	–	91.7/96.0	79.7/87.3	79.4/85.6	81.2/86,3	58.2/52.7	63.2/63.7	63.9/63.4	87.6/91.0
Polyprotein	94.0/99.3	–	93.3/98.3	76.0/80.7	76.0/80.2	76.7/81.4	52.1/46.1	56.7/54.8	57.1/55.2	79.4/82.3

*Values were described as nucleotide identity (%)/amino acid identity (%)*.

### Similarity Plot Analysis

To further analyze the genetic characteristics of the complete polyprotein gene, similarity plot analysis was conducted using SimPlot 3.5.1 which compared polyprotein nucleotide sequences of SZ59, SZ171, and the human *Kobuvirus* sequence AB010145 (used as the out-group sequence) to murine *Kobuvirus* reference strain Wencheng-Rt386-2/MF352432 (used as the query sequence). SZ59 and SZ171 exhibited relatively high similarity to the query sequence in the 5′-UTR, VP3, 2A-2C, 3B-3D, and 3′-UTR regions, but less similarity in the L, VP0, VP1, 3A regions as well as a region near the start of the 3D gene (Supplementary Figure 2). A higher range of genetic variability was observed in the P1 coding region, and intergenotype recombination was not observed in the *Kobuvirus* coding regions.

## Discussion

To the best of our knowledge, this is the first study to investigate the prevalence and genomes of kobuviruses in multiple samples (fecal, lung, throat swab, and serum) from different murine rodent species (*Rattus norvegicus, Rattus tanezumi*, and *Rattus rattus*). We investigated 413 fecal samples, of which 143 (34.6%) were positive for *Kobuvirus*, with detection rates of 34.9% in *Rattus norvegicus*, 29.4% in *Rattus tanezumi*, and 0% in *Rattus rattus*. In our previous study, detection rates were 23.7% in *Rattus tanezumi*, 10.2% in *Rattus losea*, and 28.1% in *Rattus norvegicus* in southern China (26), and the prevalence in *Rattus norvegicus* was 12.6% in Guangzhou (25). MuKVs were detected in 50% of *Rattus norvegicus* fecal samples in New York in 2014 (24), and were present in 55.5% of free-living and laboratory rats (*Rattus norvegicus*) in Hungary (22). In New York (Manhattan and Queens sites) in 2018, MuKV was detected in 17.5% of *Mus musculus* samples (23), and in Vietnam the prevalence rate of MuKV in rats was 17% (17). These findings reveal that kobuviruses are prevalent among different species of murine rodents. In this report, although the prevalence of kobuviruses in fecal samples did not differ significantly between the species, the difference among seasons was significant, with the highest prevalence in winter, suggesting that murine *Kobuvirus* is prevalent throughout the year, but more attention should be paid to disease prevention and control in winter. Furthermore, both female and male rats can carry *Kobuvirus*, and there was no statistical difference between the sexes.

Previous studies suggest that kobuviruses may play a role as causative agents in gastroenteritis diseases (5), and are often found in gastrointestinal tract samples from humans and other animals (5, 9–20), suggesting that *Kobuvirus* is transmitted via the fecal-oral route or consumption of contaminated food or water. The detection of kobuviruses in sewage samples in South Africa (18) and Tunisia (35), in surface waters in Venezuela (36), in sewage and surface waters in the Netherlands (37), and in sewage and river waters in Japan (38), and a new *Kobuvirus* closely related to canine *Kobuvirus* detected in sewage samples in Japan (34) supports this contention. In the current study, only MuKV was found in rodents feces, and in phylogenetic analyses all sequences obtained clustered together with the sewage-derived *Kobuvirus* found in Nepal (33), and shared more than 86% nucleotide and 87% amino acid identities, confirming that MuKV is mainly transmitted via the fecal-oral route through contaminated water and raw and treated sewage, which is concordant with previous Tunisian, Japanese, and Dutch studies (35, 37, 38). Furthermore, we recently conducted two initial epidemiological studies investigating MuKV in murine rodent stool samples (25, 26) which also provide evidence that is congruent with this finding.

Seroepidemiologic studies in Japan, Germany, France, Spain, and Tunisia (27, 39–42) indicate general circulation of kobuviruses in human populations, suggesting an important role of *Kobuvirus* infection in gastroenteritis. However, a report of porcine viremia suggests that *Kobuvirus* can escape the gastrointestinal tract into the circulatory system in immunocompetent virus-infected hosts (28), and its high prevalence in dog and cat serum samples indicates the importance of *Kobuvirus* detection in animal serum specimens (29). Further investigations are required to confirm whether the digestive tract is the main location of *Kobuvirus* replication. Notably, kobuviruses have also been identified in other sample types. MuKV was found in feces and liver samples from *Mus musculus* (23), and in fecal, lung, brain, and heart specimens from an infected *Rattus norvegicus* (24). Interestingly, canine *Kobuvirus* was detected in brain, lung, tonsil, and liver samples from a puppy, but feces and gastrointestinal tract samples from that same puppy were negative (30). These findings confirm widespread viral dissemination in multiple tissues of animals, but also expand the potential tissue tropisms and transmission routes of kobuviruses.

In neighbor-joining phylogenetic analysis based on partial 3D or VP1 regions, the MuKV sequences isolated from *Rattus norvegicus* and *Rattus tanezumi* in the current study clustered with other rat-derived *Kobuvirus* sequences identified in China, Hungary, and the USA. In phylogenetic tree analysis based on partial 3D regions, the sequences obtained clustered tightly together in a clade according to the species, confirming that the 3D gene is conserved. Notably, however, phylogenetic analysis based on the VP1 regions indicated that such origin-specific lineages of species were not clear. Although the above results suggest that MuKV has distinctive characteristics that differ from other *Aichivirus A* viruses, the possibility of cross-species transmission in *Kobuvirus* cannot be ignored. Historical transmissions between rabbits and bats, between rodents and bats, and between cows and pigs have been reported (17, 43, 44), raising the possibility of cross-species transmission in the future; which could result in a sustained threat to public health due to increased contact between humans and animals. More evidence is required to prove cross-species transmission of *Kobuvirus*, and assess the pathogenicity posed to human health.

Amino acid mutations cause changes in protein phenotype, and are considered most deleterious. Both the 3D and VP1 regions investigated in the current study are important in the stability of the *Kobuvirus* genome, and its replication. In amino acid heterogeneity analysis, the sequences identified in the study were highly similar, and the 3D region was relatively conserved whereas the VP1 region had more amino acid deletions and substitutions. These results are concordant with a previous review indicating that VP1 is the most variable structural protein and the 3D gene is highly conserved among kobuviruses (3). Estimation of these changes in genomic regions would be helpful with respect to the characteristics of kobuviruses.

In total, 28 complete gene sequences of MuKV were successfully acquired in this study. The polyproteins of the sequences were all cleaved into 11 viral proteins, including L, P1 (VP0, VP3, and VP1), P2 (2A−2C), and P3 (3A−3D), and the predicted cleavage sites were Q/G, P/Q, Q/H, Q/S, Q/T, Q/A, Q/P, Y/V, Q/C, and A/T, according to alignment with known *Kobuvirus* cleavage sites. Comparisons of the protease cleavage sites in *Kobuvirus* sequences isolated from humans and animals indicated that the protease cleavage site sequences are highly conserved, supporting a previous review suggesting that the predicted cleavage sites are similar to those in other picornaviruses (3). As representatives of two species, SZ59 shared relatively high nucleotide and amino acid identity with SZ171 (94.0 and 99.3%, respectively), and with other full reference sequences. SZ59 and SZ171 had the highest similarity to the MuKV sequences (93.3–93.6% nucleotide and 98.1–98.3% amino acid identities), with identity values <60% similar to ovine, porcine, and bovine sequences. In similarity plot analysis, SZ59 and SZ171 had lower similarities at the L, VP0, VP1, and 3A regions, and the region at the start of the 3D gene, indicating possible changes in these regions.

In the current study, all the lung, throat swab, and serum samples were negative for *Kobuvirus*. This may be related to an insufficient sample size, lack of representation in rat species, and geographic distribution. We also screened *Kobuvirus*-positive samples for *Rosavirus, Bocavirus*, and *Hunnivirus*, and 52, 43, and 6 samples, respectively, were co-infected with these viruses. These results are consistent with previous studies demonstrating that kobuviruses are frequently present in mixed infections with other pathogens (45, 46).

## Conclusion

The present study provides evidence of fecal-oral transmission of MuKV, and increases the knowledge of molecular characteristics and evolution of MuKV. Further epidemiological and laboratory studies are needed to verify fecal-oral transmission of MuKV.

## Data Availability Statement

The datasets presented in this study can be found in online repositories. The names of the repository/repositories and accession number(s) can be found in the article/Supplementary Material.

## Ethics Statement

The animal study was reviewed and approved by Animal Ethics and Welfare Committee of the School of Public Health, Southern Medical University.

## Author Contributions

YG and QC conceived of the project. QC obtained the funding. YG contributed to the writing of the article and analyzed the data. YG, WH, and JF performed the experiment. YG, WH, JF, YL, and HH collected the samples. All authors have read and approved the manuscript for publication. All authors contributed to the article and approved the submitted version.

## Funding

This work was supported by the National Natural Science Foundation of China (Grant No. 81973107). The funders had no role in the study design, data collection and analysis, decision to publish, or preparation of the manuscript.

## Conflict of Interest

The authors declare that the research was conducted in the absence of any commercial or financial relationships that could be construed as a potential conflict of interest.

## Publisher's Note

All claims expressed in this article are solely those of the authors and do not necessarily represent those of their affiliated organizations, or those of the publisher, the editors and the reviewers. Any product that may be evaluated in this article, or claim that may be made by its manufacturer, is not guaranteed or endorsed by the publisher.

## References

[1] Zell R. *Picornaviridae*-the ever-growing virus family. Arch Virol. (2018) 163:299–317. 10.1007/s00705-017-3614-8 29058149

[2] Akagami M Ito M Niira K Kuroda M Masuda T Haga K. Complete genome analysis of porcine kobuviruses from the feces of pigs in Japan. Virus Genes. (2017) 53:593–602. 10.1007/s11262-017-1464-9 28484931

[3] Reuter G Boros A Pankovics P. Kobuviruses - a comprehensive review. Rev Med Virol. (2011) 21:32–41. 10.1002/rmv.677 21294214

[4] Yamashita T Kobayashi S Sakae K Nakata S Chiba S Ishihara Y. Isolation of cytopathic small round viruses with BS-C-1 cells from patients with gastroenteritis. J Infect Dis. (1991) 164:954–7. 10.1093/infdis/164.5.954 1658159

[5] Khamrin P Maneekarn N Okitsu S Ushijima H. Epidemiology of human and animal kobuviruses. Virusdisease. (2014) 25:195–200. 10.1007/s13337-014-0200-5 25674585 PMC4188179

[6] Yamashita T Ito M Kabashima Y Tsuzuki H Fujiura A Sakae K. Isolation and characterization of a new species of *Kobuvirus* associated with cattle. J Gen Virol. (2003) 84(Pt 11):3069–77. 10.1099/vir.0.19266-0 14573811

[7] Abi KM Zhang Q Jing ZZ Tang C. First detection and molecular characteristics of caprine *Kobuvirus* in goats in China. Infect Genet Evol. (2020) 85:104566. 10.1016/j.meegid.2020.104566 32976973

[8] Reuter G Boldizsár A Kiss I Pankovics P. Candidate new species of *Kobuvirus* in porcine hosts. Emerg Infect Dis. (2008) 14:1968–70. 10.3201/eid1412.080797 19046542 PMC2634637

[9] Li L Pesavento PA Shan T Leutenegger CM Wang C Delwart E. Viruses in diarrhoeic dogs include novel kobuviruses and sapoviruses. J Gen Virol. (2011) 92(Pt 11):2534–41. 10.1099/vir.0.034611-0 21775584 PMC3352364

[10] Chung JY Kim SH Kim YH Lee MH Lee KK Oem JK. Detection and genetic characterization of feline kobuviruses. Virus Genes. (2013) 47:559–62. 10.1007/s11262-013-0953-8 23963764 PMC7088707

[11] Di Martino B Di Profio F Melegari I Robetto S Di Felice E Orusa R. Molecular evidence of kobuviruses in free-ranging red foxes (*Vulpes vulpes*). Arch Virol. (2014) 159:1803–6. 10.1007/s00705-014-1975-9 24452667 PMC7086952

[12] Smits SL Raj VS Oduber MD Schapendonk CM Bodewes R Provacia L. Metagenomic analysis of the ferret fecal viral flora. PLoS ONE. (2013) 8:e71595. 10.1371/journal.pone.0071595 23977082 PMC3748082

[13] Lee MH Jeoung HY Lim JA Song JY Song DS An DJ. *Kobuvirus* in South Korean black goats. Virus Genes. (2012) 45:186–9. 10.1007/s11262-012-0745-6 22528642

[14] Pankovics P Boros Á Bíró H Horváth KB Phan TG Delwart E. Novel picornavirus in domestic rabbits (*Oryctolagus cuniculus* var. domestica). Infect Genet Evol. (2016) 37:117–22. 10.1016/j.meegid.2015.11.012 26588888 PMC7172602

[15] Li L Victoria JG Wang C Jones M Fellers GM Kunz TH. Bat guano virome: predominance of dietary viruses from insects and plants plus novel mammalian viruses. J Virol. (2010) 84:6955–65. 10.1128/JVI.00501-10 20463061 PMC2898246

[16] Phan TG Kapusinszky B Wang C Rose RK Lipton HL Delwart EL. The fecal viral flora of wild rodents. PLoS Pathog. (2011) 7:e1002218. 10.1371/journal.ppat.1002218 21909269 PMC3164639

[17] Lu L Van Dung N Ivens A Bogaardt C O'Toole A Bryant JE. Genetic diversity and cross-species transmission of kobuviruses in Vietnam. Virus Evol. (2018) 4:vey002. 10.1093/ve/vey002 29449965 PMC5810437

[18] Onosi O Upfold NS Jukes MD Luke GA Knox C. The first molecular detection of Aichi virus 1 in raw sewage and mussels collected in South Africa. Food Environ Virol. (2019) 11:96–100. 10.1007/s12560-018-9362-4 30560489

[19] Adams MJ King AM Carstens EB. Ratification vote on taxonomic proposals to the International Committee on Taxonomy of Viruses 2013. Arch Virol. (2013) 158:2023–30. 10.1007/s00705-013-1688-5 23580178

[20] Zell R Delwart E Gorbalenya AE Hovi T King AMQ Knowles NJ. ICTV virus taxonomy profile: *Picornaviridae*. J Gen Virol. (2017) 98:2421–2. 10.1099/jgv.0.000911 28884666 PMC5725991

[21] Farkas T Fey B Keller G Martella V Egyed L. Molecular detection of novel astroviruses in wild and laboratory mice. Virus Genes. (2012) 45:518–25. 10.1007/s11262-012-0803-0 22899339

[22] Boros Á Orlovácz K Pankovics P Szekeres S Földvári G Fahsbender E. Diverse picornaviruses are prevalent among free-living and laboratory rats (*Rattus norvegicus*) in Hungary and can cause disseminated infections. Infect Genet Evol. (2019) 75:103988. 10.1016/j.meegid.2019.103988 31377399

[23] Williams SH Che X Garcia JA Klena JD Lee B Muller D. Viral diversity of house mice in New York City. mBio. (2018) 9:e01354-17. 10.1128/mBio.01354-17 29666290 PMC5904411

[24] Firth C Bhat M Firth MA Williams SH Frye MJ Simmonds P. Detection of zoonotic pathogens and characterization of novel viruses carried by commensal *Rattus norvegicus* in New York City. mBio. (2014) 5:e01933-14. 10.1128/mBio.01933-14 25316698 PMC4205793

[25] You FF Zhang MY He H He WQ Li YZ Chen Q. Kobuviruses carried by *Rattus norvegicus* in Guangdong, China. BMC Microbiol. (2020) 20:94. 10.1186/s12866-020-01767-x 32295529 PMC7161169

[26] Zhang M You F Wu F He H Li Q Chen Q. Epidemiology and genetic characteristics of murine *Kobuvirus* from faecal samples of *Rattus losea, Rattus tanezumi* and *Rattus norvegicus* in southern China. J Gen Virol. (2021) 102:001646. 10.1099/jgv.0.001646 34486970 PMC8567428

[27] Yamashita T Sakae K Ishihara Y Isomura S Utagawa E. Prevalence of newly isolated, cytopathic small round virus (Aichi strain) in Japan. J Clin Microbiol. (1993) 31:2938–43. 10.1128/jcm.31.11.2938-2943.1993 8263178 PMC266163

[28] Reuter G Kecskémeti S Pankovics P. Evolution of porcine *Kobuvirus* infection, Hungary. Emerg Infect Dis. (2010) 16:696–8. 10.3201/eid1604.090937 20350391 PMC3321945

[29] Carmona-Vicente N Buesa J Brown PA Merga JY Darby AC Stavisky J. Phylogeny and prevalence of kobuviruses in dogs and cats in the UK. Vet Microbiol. (2013) 164:246–52. 10.1016/j.vetmic.2013.02.014 23490561 PMC7127238

[30] Ribeiro J Headley SA Diniz JA Pereira AH Lorenzetti E Alfieri AA. Extra-intestinal detection of canine *Kobuvirus* in a puppy from Southern Brazil. Arch Virol. (2017) 162:867–72. 10.1007/s00705-016-3164-5 27888408 PMC7086620

[31] Kocher TD Thomas WK Meyer A Edwards SV Pääbo S Villablanca FX. Dynamics of mitochondrial DNA evolution in animals: amplification and sequencing with conserved primers. Proc Natl Acad Sci USA. (1989) 86:6196–200. 10.1073/pnas.86.16.6196 2762322 PMC297804

[32] Reuter G Boldizsár A Pankovics P. Complete nucleotide and amino acid sequences and genetic organization of porcine *Kobuvirus*, a member of a new species in the genus *Kobuvirus*, family *Picornaviridae*. Arch Virol. (2009) 154:101–8. 10.1007/s00705-008-0288-2 19096904

[33] Ng TF Marine R Wang C Simmonds P Kapusinszky B Bodhidatta L. High variety of known and new RNA and DNA viruses of diverse origins in untreated sewage. J Virol. (2012) 86:12161–75. 10.1128/JVI.00869-12 22933275 PMC3486453

[34] Yamashita T Adachi H Hirose E Nakamura N Ito M Yasui Y. Molecular detection and nucleotide sequence analysis of a new Aichi virus closely related to canine *Kobuvirus* in sewage samples. J Med Microbiol. (2014) 63(Pt 5):715–20. 10.1099/jmm.0.070987-0 24523156

[35] Sdiri-Loulizi K Hassine M Aouni Z Gharbi-Khelifi H Sakly N Chouchane S. First molecular detection of Aichi virus in sewage and shellfish samples in the Monastir region of Tunisia. Arch Virol. (2010) 155:1509–13. 10.1007/s00705-010-0744-7 20607319

[36] Alcalá A Vizzi E Rodríguez-Díaz J Zambrano JL Betancourt W Liprandi F. Molecular detection and characterization of Aichi viruses in sewage-polluted waters of Venezuela. Appl Environ Microbiol. (2010) 76:4113–5. 10.1128/AEM.00501-10 20418428 PMC2893485

[37] Lodder WJ Rutjes SA Takumi K de Roda Husman AM. Aichi virus in sewage and surface water, the Netherlands. Emerg Infect Dis. (2013) 19:1222–30. 10.3201/eid1908.130312 23876456 PMC3739534

[38] Kitajima M Haramoto E Phanuwan C Katayama H. Prevalence and genetic diversity of Aichi viruses in wastewater and river water in Japan. Appl Environ Microbiol. (2011) 77:2184–7. 10.1128/AEM.02328-10 21257803 PMC3067340

[39] Goyer M Aho LS Bour JB Ambert-Balay K Pothier P. Seroprevalence distribution of Aichi virus among a French population in 2006-2007. Arch Virol. (2008) 153:1171–4. 10.1007/s00705-008-0091-0 18446423

[40] Oh DY Silva PA Hauroeder B Diedrich S Cardoso DD Schreier E. Molecular characterization of the first Aichi viruses isolated in Europe and in South America. Arch Virol. (2006) 151:1199–206. 10.1007/s00705-005-0706-7 16421634

[41] Ribes JM Montava R Téllez-Castillo CJ Fernández-Jiménez M Buesa J. Seroprevalence of Aichi virus in a Spanish population from 2007 to 2008. Clin Vaccine Immunol. (2010) 17:545–9. 10.1128/CVI.00382-09 20164249 PMC2849335

[42] Sdiri-Loulizi K Ambert-Balay K Gharbi-Khelifi H Sakly N Hassine M Chouchane S. Molecular epidemiology of norovirus gastroenteritis investigated using samples collected from children in Tunisia during a four-year period: detection of the norovirus variant GGII.4 Hunter as early as January 2003. J Clin Microbiol. (2009) 47:421–9. 10.1128/JCM.01852-08 19109464 PMC2643701

[43] Allocati N Petrucci AG Di Giovanni P Masulli M Di Ilio C De Laurenzi V. Bat-man disease transmission: zoonotic pathogens from wildlife reservoirs to human populations. Cell Death Discov. (2016) 2:16048. 10.1038/cddiscovery.2016.48 27551536 PMC4979447

[44] Khamrin P Maneekarn N Hidaka S Kishikawa S Ushijima K Okitsu S. Molecular detection of *Kobuvirus* sequences in stool samples collected from healthy pigs in Japan. Infect Genet Evol. (2010) 10:950–4. 10.1016/j.meegid.2010.06.001 20547246

[45] Yang Z Jin W Zhao Z Lin W Zhang D Yu E. Genetic characterization of porcine *Kobuvirus* and detection of coinfecting pathogens in diarrheic pigs in Jiangsu Province, China. Arch Virol. (2014) 159:3407–12. 10.1007/s00705-014-2204-2 25119679

[46] Zhao ZP Yang Z Lin WD Wang WY Yang J Jin WJ. The rate of co-infection for piglet diarrhea viruses in China and the genetic characterization of porcine epidemic diarrhea virus and porcine *Kobuvirus*. Acta Virol. (2016) 60:55–61. 10.4149/av_2016_01_55 26982468

