# Supplementary material for: Epidemiological Evidence for Fecal-Oral Transmission of Murine Kobuvirus
Source: Front Public Health. 2022 Apr 19;10:865605. doi: 10.3389/fpubh.2022.865605 (PMC9062591; doi:10.3389/fpubh.2022.865605)
Supplement: Supplementary file 1 [file Data_Sheet_1.pdf]

Supplementary Material

1 Supplementary Figures and Tables

1.1 Supplementary Figures

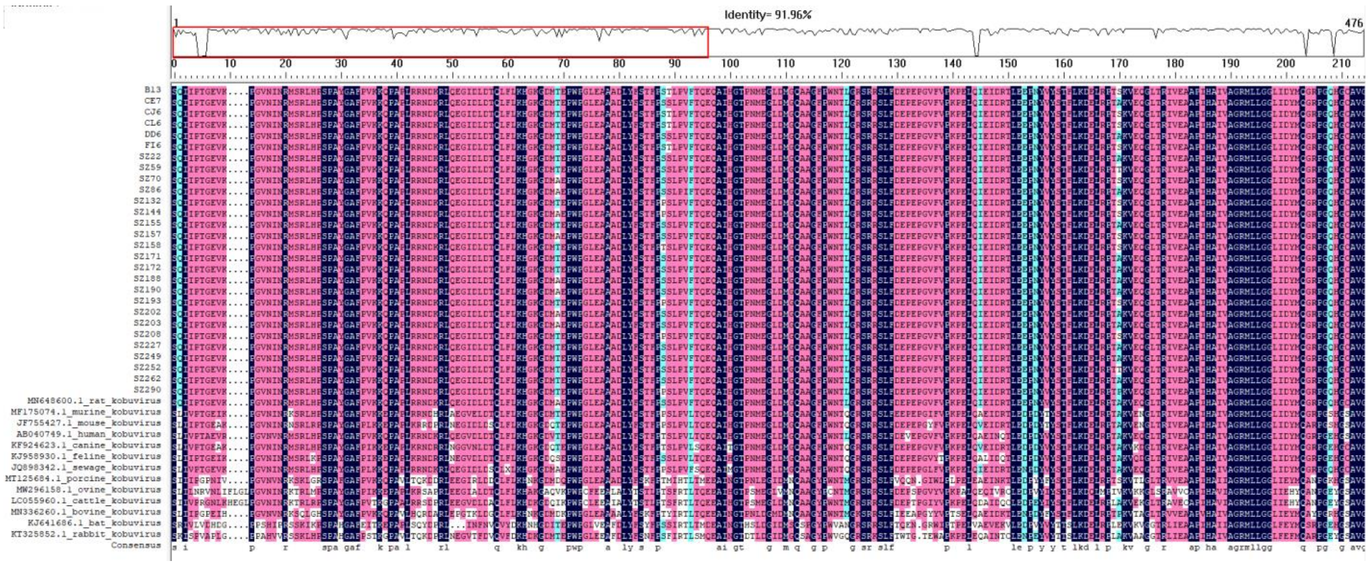

(A)

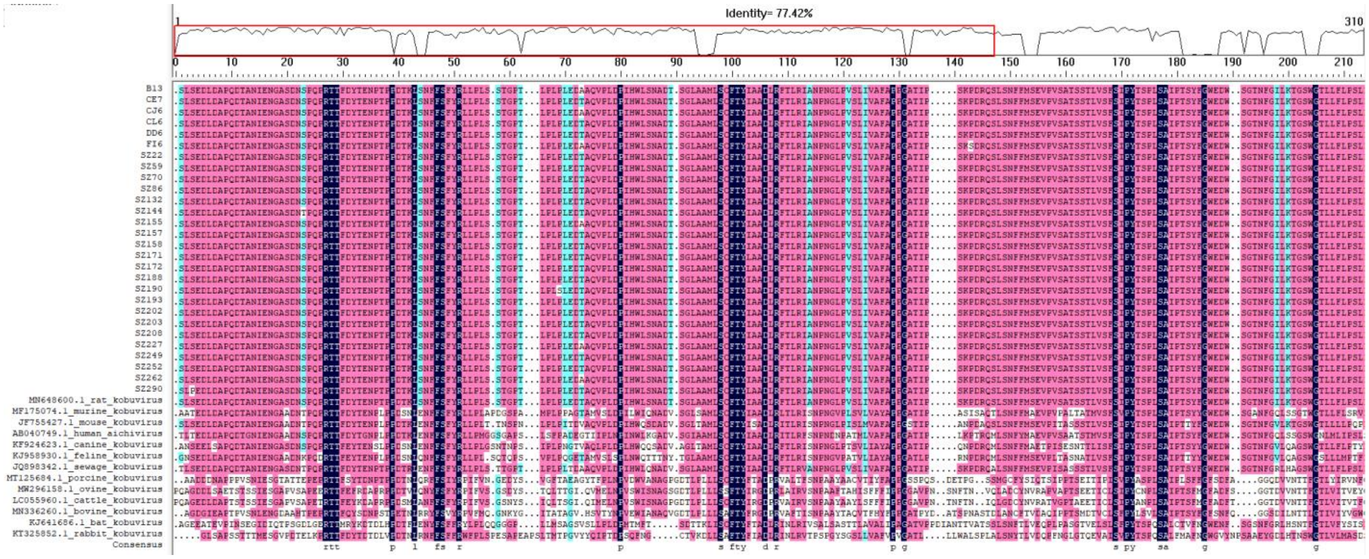

(B)

Supplementary Figure 1. Comparison of the deduced protein amino acid sequences of the study strains and the reference strains. (A) Comparison of the deduced 3D protein amino acid sequences (468 aa). (B) Comparison of the deduced VP1 amino acid sequences (277 aa). Color marks amino acid substitution; dot (.) indicates amino acid deletion.

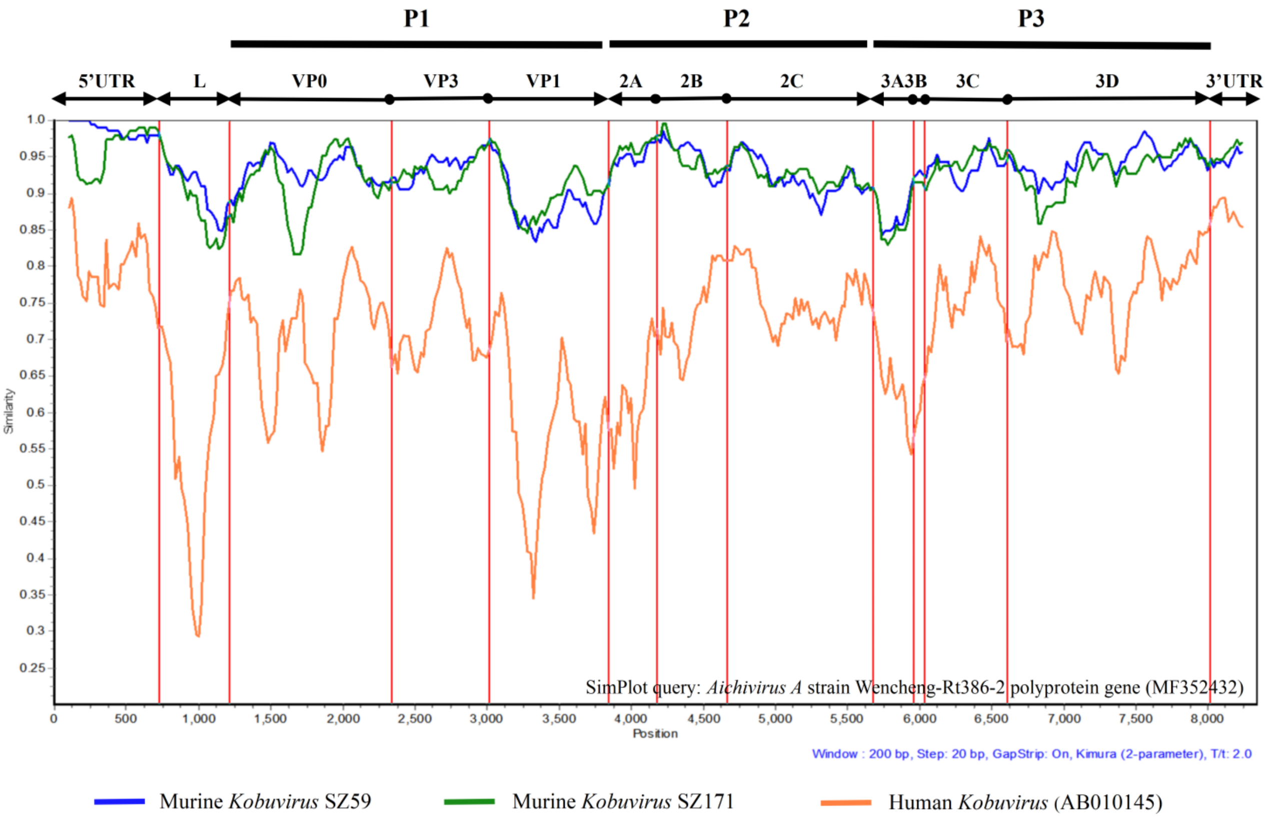

Supplementary Figure 2. Similarity plot analysis of the complete genomes of SZ59 (blue line) and SZ171 (green line) identified in this study. The human *Kobuvirus* AB010145 (orange line) was used as an out-group sequence, and *Aichivirus A* strain Wencheng-Rt386-2 (MF352432) as a query sequence, using a Kimura (2-parameter) model with a sliding window of 200 nt and a moving step size of 20 nt in Simplot 3.5.1 software.

1.2 Supplementary Tables

Supplementary Table 1. Information for primer sequences.

Number	Primer Name	Sequences (5’-3’)	Target fragment(bp)
1	UNIV-kobu-F	TGGAYTACAARTGTTTTGATGC	216
	UNIV-kobu-R	ATGTTGTTRATGATGGTGTTGA	
2	Kobu-F14	TCGGCCCCCTCACCCTCTTTTC	597
	Kobu-R610	GTCATCCCTTCCAGGGCGTTGG	
3	Kobu-F262	CAGCCTGACGTGTCACAGGCTG	514
	Kobu-R775	GCAGCAACAGCAGAGCGCACAG	
4	Kobu-F658	GCGTAAGGTTCAAGTGC	1000
	Kobu-R1657	CGGTTGCCTGAGGAGAC	
5	Kobu-F1536	GGCATCGAAGGTGCTGGTAAGG	790
	Kobu-R2325	GTCACGTAGATGGAGCAGGA	
6	Kobu-F2238	ACCACCAGTGGGCTCCATGC	841
	Kobu-R3078	GCAGTRTCCTGCGGAGCATC	
7	Kobu-F2556	TCCCTCAACTGGACTATGGC	727
	Kobu-R3282	AGCCAATGGATRGGGTCTAG	
8	Kobu-VP1-os	GTCCAYTGGAARACAGTGGACAT	831
	Kobu-VP1-oa	ACCGACTCCRAGRGCCARTG	
	Kobu-VP1-is	TCCTTYAACGTYCGYYTCATGC	
	Kobu-VP1-ia	CARTGAGAAGCCGGTGTTGGG	
9	Kobu-F3929	TGATGGTAGCCTGAAGCAGATC	963
	Kobu-R4891	GCCAATATGGAATCYGAGTAGT	
10	Kobu-F4742	CAATGACGGTGCACTCGCCATG	792
	Kobu-R5533	TTGGACTGGGTTCCRGGGATGG	
11	Kobu-F5316	CCCAACCTGGTTTCCTCAGC	885
	Kobu-R6200	GTGGGGACAATGAGGTAGCG	
12	Kobu-F5748	GAGGCTGAGCCCCGGGAAAT	781
	Kobu-R6528	TCCGTGACAAGCGGGGAACC	
13	Kobu-F6426	GTCCGCATCTCTGACAACAG	808
	Kobu-R7233	AGACCACCGAGGAGCATCCG	
14	Kobu-F7085	GTCGTTCGCTCTTTGAT	851
	Kobu-R7935	CACTGATTGCTCGTAGGT	
15	Kobu-F7818	CCTGATGACAAGCGTCCATTC	472
	Kobu-R8289	AAGCTCAAAGACWACCCTGGC	

Supplementary Table 2.

Protease-cleavage sites of Murine *Kobuvirus* (MuKV) sequences obtained in this study and several *Kobuvirus* reference sequences from other species.

		Cleavage between									
		**L/VP0**	**VP0/VP3**	**VP3/VP1**	**VP1/2A**	**2A/2B**	**2B/2C**	**2C/3A**	**3A/3B**	**3B/3C**	**3C/3D**
	**SZ-B13**	QLQRQ/G	RYVAP/QH	ALSSQ/SL	QRPTY/VH	GIRRQ/GL	TLEHQ/GL	LIKRQ/GN	PSSSQ/AA	HIQRQ/GI	ATTHQ/S
	**SZ-CE7**	QLQRQ/G	RYVAP/QH	ALSSQ/SL	QRPTY/VH	GIRRQ/GL	TLEHQ/GL	LIKRQ/GN	PSSSQ/AA	HIQRQ/GI	ATTHQ/S
	**SZ-CJ6**	QLQRQ/G	RYVAP/QH	ALSSQ/SL	QRPTY/VH	GIRRQ/GL	TLEHQ/GL	LIKRQ/GN	PSSSQ/AA	HIQRQ/GI	ATTHQ/S
	**SZ-CL6**	QLQRQ/G	RYVAP/QH	ALSSQ/SL	QRPTY/VH	GIRRQ/GL	TLEHQ/GL	LIKRQ/GN	PSSSQ/AA	HIQRQ/GI	ATTHQ/S
	**SZ-DD6**	QLQRQ/G	RYVAP/QH	ALSSQ/SL	QRPTY/VH	GIRRQ/GL	TLEHQ/GL	LIKRQ/GN	PSSSQ/AA	HIQRQ/GI	ATTHQ/S
	**SZ-FI6**	QLQRQ/G	RYVAP/QH	ALSSQ/SL	QRPTY/VH	GIRRQ/GL	TLEHQ/GL	LIKRQ/GR	PSSSQ/AA	HIQRQ/GI	ATTHQ/S
	**SZ22**	QLQRQ/G	RYVTP/QH	ALSSQ/SL	QRPTY/VH	GIRRQ/GL	TLEHQ/GL	LIKRQ/GR	PSSSQ/AA	HIQRQ/GI	ATTHQ/S
	**SZ59**	QLQRQ/G	RYVAP/QH	ALSSQ/SL	QRPTY/VH	GIRRQ/GL	TLEHQ/GL	LIKRQ/GR	PSSTQ/AA	HIQRQ/GI	ATTHQ/S
**Rat**	**SZ70**	QLQRQ/G	RYVAP/QH	ALSSQ/SL	QRPTY/VH	GIRRQ/GL	TLEHQ/GL	LIKRQ/GR	PTSSQ/AA	HIQRQ/GI	ATTHQ/S
	**SZ86**	QLQRQ/G	RYVAP/QH	ALSSQ/SL	QRPTY/VH	GIRRQ/GL	TLEHQ/GL	LIKRQ/GR	PSSSQ/AA	HIQRQ/GI	ATTHQ/S
	**SZ132**	QLQRQ/G	RYVAP/QH	ALSSQ/SL	QRPTY/VH	GIRRQ/GL	TLEHQ/GL	LIKRQ/GR	PSSTQ/AA	HIQRQ/GI	ATTHQ/S
	**SZ144**	QLQRQ/G	RYVAP/QH	ALSSQ/SL	QRPTY/VH	GIRRQ/GL	TLEHQ/GL	LIKRQ/GR	PSSSQ/AA	HIQRQ/GI	ATTHQ/S
	**SZ155**	QLQRQ/G	RYVAP/QH	ALSSQ/SL	QRPTY/VH	GIRRQ/GL	TLEHQ/GL	LIKRQ/GR	PSSSQ/AA	HIQRQ/GI	ATTHQ/S
	**SZ157**	QLQRQ/G	RYVAP/QH	ALSSQ/SL	QRPTY/VH	GIRRQ/GL	TLEHQ/GL	LIKRQ/GR	PTSSQ/AA	HIQRQ/GI	ATTHQ/S
	**SZ158**	QLQRQ/G	RYVAP/QH	ALSSQ/SL	QRPTY/VH	GIRRQ/GL	TLEHQ/GL	LIKRQ/GR	PSSSQ/AA	HIQRQ/GI	ATTHQ/S
	**SZ171**	QLQRQ/G	RYVAP/QH	ALSSQ/SL	QRPTY/VH	GIRRQ/GL	TLEHQ/GL	LIKRQ/GR	PSSSQ/AA	HIQRQ/GI	ATTHQ/S
	**SZ172**	QLQRQ/G	RYVAP/QH	ALSSQ/SL	QRPTY/VH	GIRRQ/GL	TLEHQ/GL	LIKRE/GR	PSSSQ/AA	HIQRQ/GI	ATTHQ/S
	**SZ188**	QLQRQ/G	RYVAP/QH	ALSSQ/SL	QRPTY/VH	GIRRQ/GL	TLEHQ/GL	LIKRQ/GR	PSSSQ/AA	HIQRQ/GI	ATTHQ/S

	**SZ190**	QLQRQ/G	RYVAP/QH	ALSSQ/SL	QRPTY/VH	GIRRQ/GL	TLEHQ/GL	LIKRQ/GR	PSSSQ/AA	HIQRQ/GI	ATTHQ/S
	**SZ193**	QLQRQ/G	RYVAP/QH	ALSSQ/SL	QRPTY/VH	GIRRQ/GL	TLEHQ/GL	LIKRQ/GR	PSSTQ/AA	HIQRQ/GI	ATTHQ/S
	**SZ202**	QLQRQ/G	RYVAP/QH	ALSSQ/SL	QRPTY/VH	GIRRQ/GL	TLEHQ/GL	LIKRQ/GR	PSSSQ/AA	HIQRQ/GI	ATTHQ/S
	**SZ203**	QLQRQ/G	RYVAP/QH	ALSSQ/SL	QRPTY/VH	GIRRQ/GL	TLEHQ/GL	LIKRQ/GR	PSSSQ/AA	HIQRQ/GI	ATTHQ/S
	**SZ208**	QLQRQ/G	RYVAP/QH	ALSSQ/SL	QRPTY/VH	GIRRQ/GL	TLEHQ/GL	LIKRQ/GR	PSSSQ/AA	HIQRQ/GI	ATTHQ/S
	**SZ227**	QLQRQ/G	RYVAP/QH	ALSSQ/SL	QRPTY/VH	GIRRQ/GL	TLEHQ/GL	LIKRQ/GR	PSSSQ/AA	HIQRQ/GI	ATTHQ/S
	**SZ249**	QLQRQ/G	RYVAP/QH	ALSSQ/SL	QRPTY/VH	GIRRQ/GL	TLEHQ/GL	LIKRQ/GR	PSSAQ/AA	HIQRQ/GI	ATTHQ/S
	**SZ252**	QLQRQ/G	RYVAP/QH	ALSSQ/SL	QRPTY/VH	GIRRQ/GL	TLEHQ/GL	LIKRQ/GN	PSSSQ/AA	HIQRQ/GI	ATTHQ/S
	**SZ262**	QLQRQ/G	RYVAP/QH	ALSSQ/SL	QRPTY/VH	GIRRQ/GL	TLEHQ/GL	LIKRQ/GR	PSSSQ/AA	HIQRQ/GI	ATTHQ/S
	**SZ290**	QLQRQ/G	RYVAP/QH	ALSSQ/SL	QRPTY/VH	GIRRQ/GL	TLEHQ/GL	LIKRQ/GR	PSSSQ/AA	HIQRQ/GI	ATTHQ/S
	**MN116647**	QLQRQ/G	RYVAP/QH	ALSSQ/TL	QRPTY/VH	GIRRQ/GL	TLEHQ/GL	LIKRQ/GN	PSSSQ/AA	HIQRQ/GI	ATTHQ/S
**Human**	**AB010145**	NLQRQ/G	RYLAP/QH	ALTSQ/TL	QRPTY/VH	GIRRQ/GL	TLEPQ/GL	LIRRQ/GN	PQEPQ/AA	HIQRQ/GI	ATTQQ/SL
**Feline**	**KJ958930**	QIKPQ/GN	YVAPQ/HW	ALSSQ/GN	VIKRQ/GA	GIKRQ/GL	TLEPQ/GL	LIKRQ/GR	QSAAQ/A	HIQRQ/GL	ATTQQ/SL
**Canine**	**MH747478**	QIKPQ/GN	YVAPQ/HW	ALTSQ/AN	AVVKQ/G	GIKRQ/GL	TLEPQ/GL	LIKRQ/GR	KSETQ/AA	HIQRQ/GL	ATTHQ/SL
**Ovine**	**MW29615**	PVERQ/GT	MVARQ/H	SLALQ/PQ	LARRQ/CQ	VAERQ/G	GVESQ/GL	VRQGA/T	EGVEQ/GA	PVQRQ/GC	ASQHQ/SL
**Porcine**	**KC204684**	QIVRQ/GN	QVQKQ/HW	GFQIQ/AA	RIVRQ/CQ	LVVRQ/GL	TVEHQ/G	LVKRQ/G	PDKTQ/GA	SVVRQ/SL	AIDQQ/SI
**Bovine**	**MN336260**	NIVTQ/GS	HVTKQ/HW	TLALQ/A	MVRRQ/C	RITRQ/GL	AVEPQ/GV	LIKRQ/GA	PDEPQ/AA	GVVRQ/A	ATECQ/SL
**Untreated**	**JQ898342**	-	RYVAP/QH	ALSSQ/TL	AVVRQ/GA	GIRRQ/GL	VLKHQ/GL	LIKRQ/GN	PESSQ/AA	HIQRQ/GI	ALTQQ/SL
